# PCA whitening improves the illumination tolerance for visual place recognition with Fourier signatures

**DOI:** 10.1038/s41598-026-38929-2

**Published:** 2026-02-10

**Authors:** Lars Offermann

**Affiliations:** Faculty of Technology, Bielefeld University, 33615 Bielefeld, Germany

**Keywords:** Engineering, Mathematics and computing, Optics and photonics

## Abstract

In vision-based mobile robotics, retrieving the image of a place that looks most similar to the current camera view can be used to localize a robot in a familiar environment. This technique is referred to as *visual place recognition (VPR)*. A key challenge of VPR are appearance changes, e.g. due to differences in illumination. In this work, we focus on Fourier signatures as an especially efficient VPR method. Fourier signatures describe panoramic images by a subset of the amplitudes of their frequency spectrum. By representing the panoramic image in the frequency domain and discarding phase information, the resulting signature of a place becomes invariant to cyclic shifts along the horizontal axis, corresponding to a rotation of the mobile robot. We show that whitening these signatures with Principal Component Analysis (PCA) substantially improves the robustness against illumination changes and attribute the improvements to the combination of scaling and decorrelation effects provided by PCA whitening. Comparing Fourier signatures with AnyLoc, a modern deep-learning-based image description method, we observe competitive VPR quality at substantially lower computational cost.

## Introduction

A mobile robot can localize itself in a mapped area by finding a recorded image of a visited place that is most similar to the current view, a technique known as Visual Place Recognition (VPR). The reliability of VPR is challenged when the view of a place changes, e.g. due to different illumination.

Following Masone and Caputo^1^, approaches to VPR are commonly implemented in three phases. First, all images of visited places and the current view are transformed into a compact vector representation. This allows for fast similarity checks, but with possibly low precision. We refer to this process as *global descriptor extraction* and the vector representation as a *descriptor*. Second, the descriptors of visited places are ranked by their similarity to the current view, and a fixed number of the top $K$ candidates is selected. Third, these candidates are refined by more discriminative, but computationally expensive algorithms.

In this work, we focus on Fourier signatures^2^ as a specific implementation of global descriptor extraction for panoramic images. In the following, we refer to the corresponding compact image representation as a *Fourier signature* or *descriptor*.

**Fig. 1:**
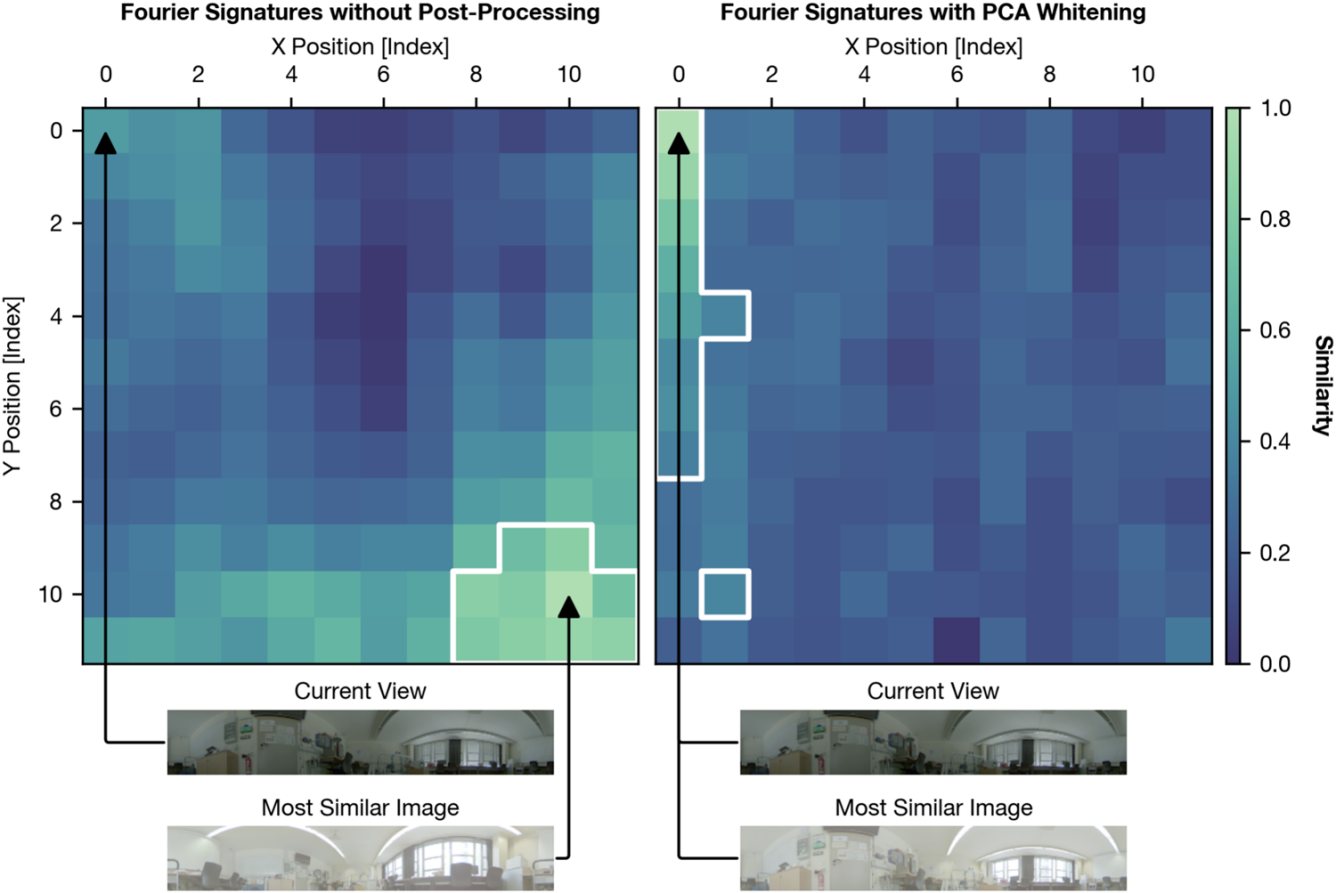
Example application of Fourier signatures without post-processing (left) and with PCA whitening applied (right) for the setting “Robotics Lab” of the grid-based image dataset introduced in “Datasets”. For both methods, we show the $S_{L1}$ similarity (see “Similarity functions”) between the descriptor of the dimly lit current view at grid index (0,0) and all the descriptors of brightly illuminated views at all other grid points. Similarity values are normalized for each of the two methods to the range [0,1] and shown in the heatmaps. The top $K=10$ candidates are marked with a white border, and the most similar image is shown on the bottom.

The implementation of Fourier signatures requires panoramic images with a full surround view as well as planar motion and negligible camera tilt between capture positions. To this end, we use the panoramic image datasets of home and office environments introduced in our previous work^3^, which capture multiple illumination conditions in each scene. These images were recorded on ground level by a mobile robot with an upward-facing fisheye camera. Fisheye images were then mapped from spherical coordinates to an equirectangular image format, such that azimuth and elevation in camera coordinates correspond to horizontal and vertical image coordinates, respectively. For example images, see Fig. 1.

A Fourier signature is then created by splitting a panoramic image along the vertical axis into rings. A ring is a section of the panoramic image that has reduced vertical height but extends to the full width and therefore captures the full 360$^\circ$ field of view. For each ring, a fixed number of the amplitudes that belong to the lowest frequencies is selected. The amplitudes are invariant against rotations in azimuthal direction with respect to camera coordinates, which allows place recognition regardless of the direction the robot is facing. We provide a full description of the algorithm with examples in “Fourier signatures”.

In this work, we propose to whiten Fourier signatures with Principal Component Analysis (PCA) and show that this additional post-processing step substantially improves the robustness of the method against illumination changes. Figure 1 demonstrates this improvement using an example application of Fourier signatures with and without PCA whitening. Additionally, we propose to compress the descriptors after the PCA transformation by retaining only a subset of the entries. We find that a substantial reduction of the original descriptor size is possible without loss of VPR quality. Additionally, we compare PCA whitening with standardization as a related post-processing technique and show that the additional decorrelation effect of PCA is advantageous in the tested environments. Finally, we show that the recall of Fourier signatures with PCA whitening is competitive with AnyLoc^4^, a recent global descriptor extraction method based on deep learning, if the number of retrieved images $K$ is 10 or less. Measurements of execution times provides evidence that Fourier signatures achieve these results while being more resource efficient.

The remainder of this paper starts with an overview of related work on global descriptor extraction and PCA for place recognition in “Related work”. Then, we introduce the datasets in “Datasets” and the evaluation process with the recall metric in “Evaluation with Recall@K”. Fourier signatures and their application to VPR are described in the subsequent sections, with a description of the basic algorithm in “Fourier signatures”, the extension with PCA whitening in “PCA whitening”, and the configured parameters in “Parameters”. Next, we present results for Fourier signatures with and without PCA whitening in “Results”. This section also provides the comparison of recall and execution times between Fourier signatures and AnyLoc. In “Discussion”, we show the influence of rescaling and decorrelation on the VPR quality. We close with a conclusion in “Conclusion”.

## Related work

In the following, we outline related work on global descriptor extraction and descriptor post-processing.

### Global descriptor extraction

Common approaches to global descriptor extraction build upon local image features. This assumes an image is characterized by the occurrence of salient, locally restricted patterns, e.g. gradients, wavelets, textures, or simple shapes.

A possible solution to creating a corresponding global descriptor is to analyze the distribution of detected local features in the image. Examples include the use of a histogram of gradients (HOG^5^) or a histogram of Gabor filters (GIST^6^).

Another possibility is to build upon existing methods that detect and describe local features, which are designed to allow recognition of the same feature after appearance changes like scaling, rotation, and illumination. Corresponding methods include SIFT^7^, ORB^8^, SuperPoint^9^, and purposefully designed parts of deep learning architectures like Convolutional Neural Networks^10,11^ and Vision Transformers^4,12,13^. Once extracted, local features must be merged into a descriptor such that both recognition of views of the same place and discriminability of views of different places are supported. This can be realized by learning the distribution of local feature descriptors using a clustering algorithm. The global descriptor then contains information about the membership of observed features to cluster centroids. Typical methods include Visual Bag-of-Words^14^, VLAD^15^, and Fisher Vectors^16^. Recently, VLAD has been used for network-based global descriptor extraction methods, e.g. NetVLAD^10^ or AnyLoc^4^. Cheng et al.^17^ demonstrate the suitability of the NetVLAD architecture to VPR with panoramic images. Another recent option is to learn local feature selection in conjunction with their description as part of a deep learning architecture, e.g. using pooling (e.g. CosPlace^11^, SelaVPR^13^) or class tokens of Vision Transformers (ViTs)^4^. We select AnyLoc^4^ as a recent ViT-based method for comparison; see “AnyLoc”.

In contrast to approaches that rely on local features, Fourier signatures assume that the image is characterized by precise frequency information, but no information where the frequencies occur in the image. In particular, Fourier signatures split a panoramic image along the vertical axis into rings and apply a 1D Fourier transform to each split. The resulting Fourier coefficients contain information about the amplitude and phase of the frequencies in the ring. Only retaining the amplitude means discarding the remaining positional information. Because the amplitude is cyclic along the horizontal image direction, Fourier signatures for a place do not change with azimuthal rotation of the camera.

Previous works^2,18,19^ have shown that frequency information can be used effectively to recognize places with panoramic images. An additional benefit is the simplicity and low computational demands compared to more demanding approaches that use local feature extraction and description or large neural networks.

### Descriptor post-processing

Previous works have explored the post-processing of global descriptors for VPR with Principal Component Analysis (PCA) and standardization.

PCA finds the principal directions and the extent of variations in a dataset as the respective eigenvectors and eigenvalues of the covariance matrix of the data, respectively. PCA whitening then decorrelates the entries of vectors in the dataset^20^ and includes three steps: First, the data are centered by subtracting the dataset mean. Second, the data points are rotated using the matrix of eigenvectors such that the principal directions align with the main coordinate axes. Third, the data are scaled to unit variance by the inverse of the root of the eigenvalues. The resulting distribution has zero mean and unit covariance. Assuming that data distributions are similar, PCA statistics can also be used to whiten descriptors for unseen data.

In terms of VPR with global image descriptors, PCA and PCA whitening are established post-processing steps with the goals of quality improvement and descriptor compression. Jégou and Chum^21^ demonstrate quality improvements with PCA whitening for descriptors created with Visual Bag-of-Words. The original VLAD and VLAD-based methods (e.g. NetVLAD and AnyLoc) use PCA to compress the otherwise large descriptor. PCA and PCA whitening have also been applied to descriptors built with neural networks, but the effectiveness depends on the particular architecture. For an overview, see the work of Masone and Caputo^1^.

An alternative post-processing step to PCA whitening is standardization, which centers and scales descriptor entries to have zero mean and unit variance. This resolves differences in scale between descriptor entries but has no decorrelation effect^20^. For VPR, Schubert et al. have shown that standardization can improve illumination tolerance for CNN-based descriptors^22^. We evaluate improvements of VPR quality for Fourier signatures with standardization as a comparison to PCA whitening.

## Methods

As means to evaluate Fourier signatures with PCA whitening, we review the image dataset, specify the partitioning into training and test data, and describe the evaluation procedure using the metric *recall@K*. Then, we introduce the Fourier signature algorithm with the proposed modifications and the successive similarity search. Lastly, we select parameters for the tested Fourier signature variants and outline our configuration of AnyLoc.

### Datasets

We use the panoramic image datasets introduced in our previous work^3^, which were recorded in 10 different home and office environments. Within these environments, the data captures typical variations of illumination. This includes the use of shutters, varying weather conditions, different daytimes, and the use of artificial illumination. In the following, a single environment is referred to as a *setting* and an instance of illumination within a setting is called a *variant*.

Images were captured on a regular grid with a spacing of 20 cm in a rectangular section of each setting. We purposefully selected obstacle-free sections with even ground. To be able to attribute differing illumination to separate variants, the environment was kept as static as possible during recording of each variant. Measurements of the 2D ground truth position of the camera were recorded with a laser measurement device (total station)^3^.

**Fig. 2:**
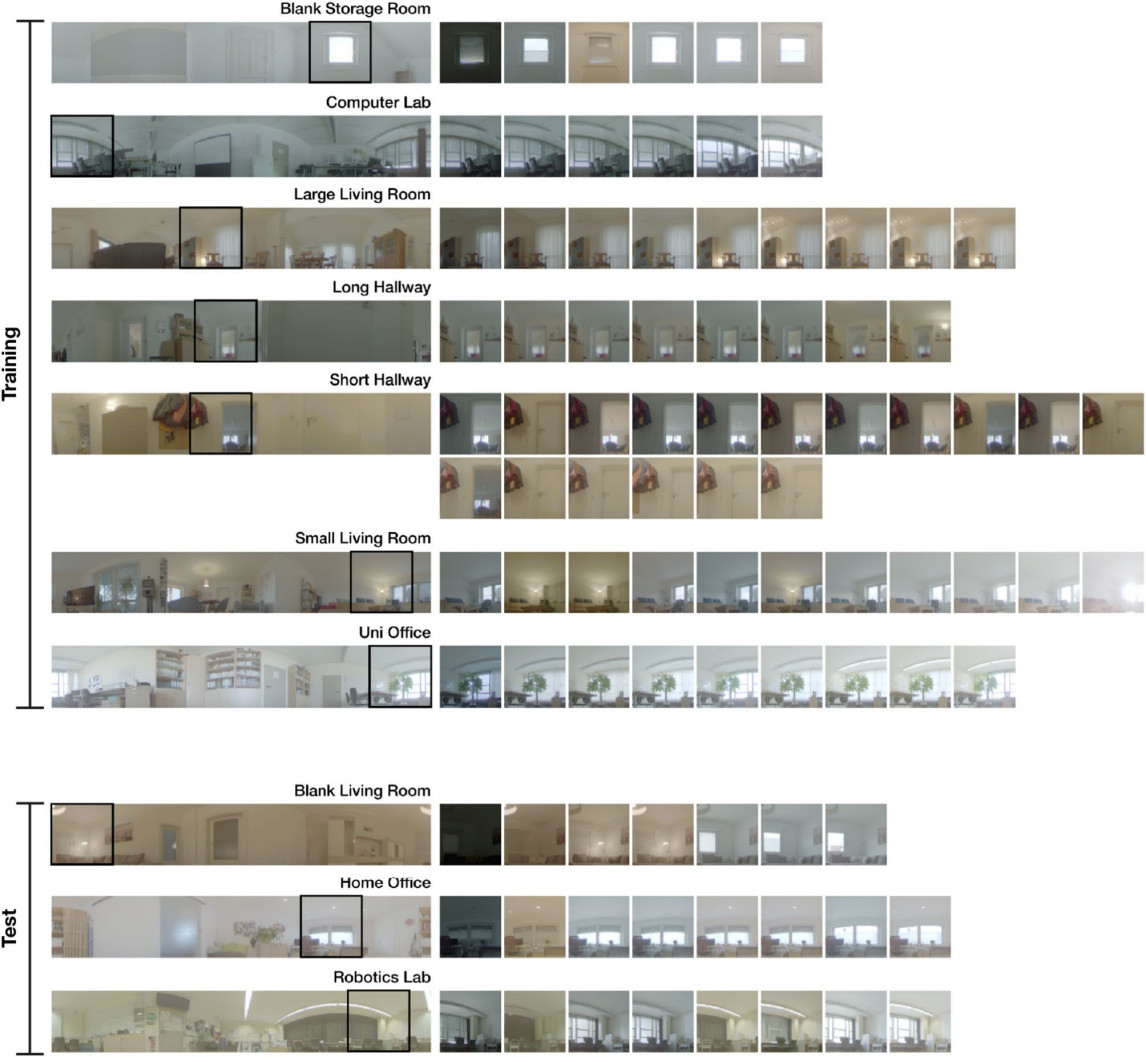
Overview of the panoramic image dataset of our previous work^3^ with partitioning into training and test splits. The leftmost column shows an overview image with a marked section. Illumination variants for this section are shown on the right.

Of the 10 available settings, we use 7 to fit the PCA and the remaining 3 to test the generalization of the learned statistics to unseen environments. An overview of settings, variants, and the dataset partitioning is shown in Fig. 2.

The dataset contains RGB color images with 99 rows and 384 columns in a high dynamic range (HDR) floating point format, which need to be processed before use. First, we convert from HDR to an 8-bit unsigned integer format as described in our previous work^3^. This includes tone mapping, clipping values to prevent artifacts in very bright regions, scaling, and discretization. Then, we remove the top 35 rows with large distortions, resulting in a final height of 64 px. As Fourier signatures require grayscale images, we convert from color using the default weighting in OpenCV 4.11^23^: $Y = 0.299R + 0.587G + 0.114B$ with $Y$ being the final gray value and $R, G, B$ describing the color values of a single pixel. Finally, we shift each image along the horizontal axis using integer values drawn from a uniform distribution to simulate a rotation of the camera in azimuthal direction. The shift is applied once before experiments are run.

### Evaluation with Recall@K

For evaluation, we only consider variant pairs with illumination changes. For any given pair, we consider all images of the first variant as visited places and successively select all images of the second variant as current views. Two images are assumed to be taken at the same place, i.e. the visited place is labeled as *positive*, if they share a grid point. We then extract Fourier signatures of the selected images of visited places and the current view. VPR is then carried out as a similarity search between the current view descriptor and all descriptors corresponding to visited places.

We evaluate Fourier signatures as part of a VPR system and assume that the similarity search is followed by a refinement step. Therefore, we use Fourier signatures to select a fixed number of $K$ candidates as the top results after sorting in descending order. While doing so, we aim to find the true positive within the top $K$ candidates for every possible current view. This quality is measured using the recall@K $R_{K,i,j} = \frac{|\textit{TP}_{i,j}|}{|P_{i,j}|}$, with $\textit{TP}_{i,j}$ as the set of true positives and $P_{i,j}$ as the set of positives for the $i$-th setting and the $j$-th variant pair.

We aggregate the recall over multiple settings by first averaging the recall@K for the $N_i$ variant pairs of the $i$-th setting, then taking the average of these setting-wise recalls: $R_{K}=\frac{1}{M} \sum _{i=1}^M(\frac{1}{N_i}\sum _{j=1}^{N_i} R_{K,i,j})$. This *macro-averaging*^24^ approach yields a recall score to which every setting contributes equally, but the weight of the individual image in the final score depends on the number of images per setting (see Table 1 in our previous work^3^). Here, images belonging to settings with fewer variants and smaller grids have a greater influence compared to images belonging to settings with more variants and larger grids. The alternative aggregation method, *micro-averaging*^24^, results in a recall score in which every image has equal weight, consequently reducing the contribution of settings with less images. The preferable choice of averaging depends on the desired application. We expect an investigated algorithm to work equally well across all test settings, which is well represented by macro-averaging.

### Fourier signatures

**Fig. 3:**
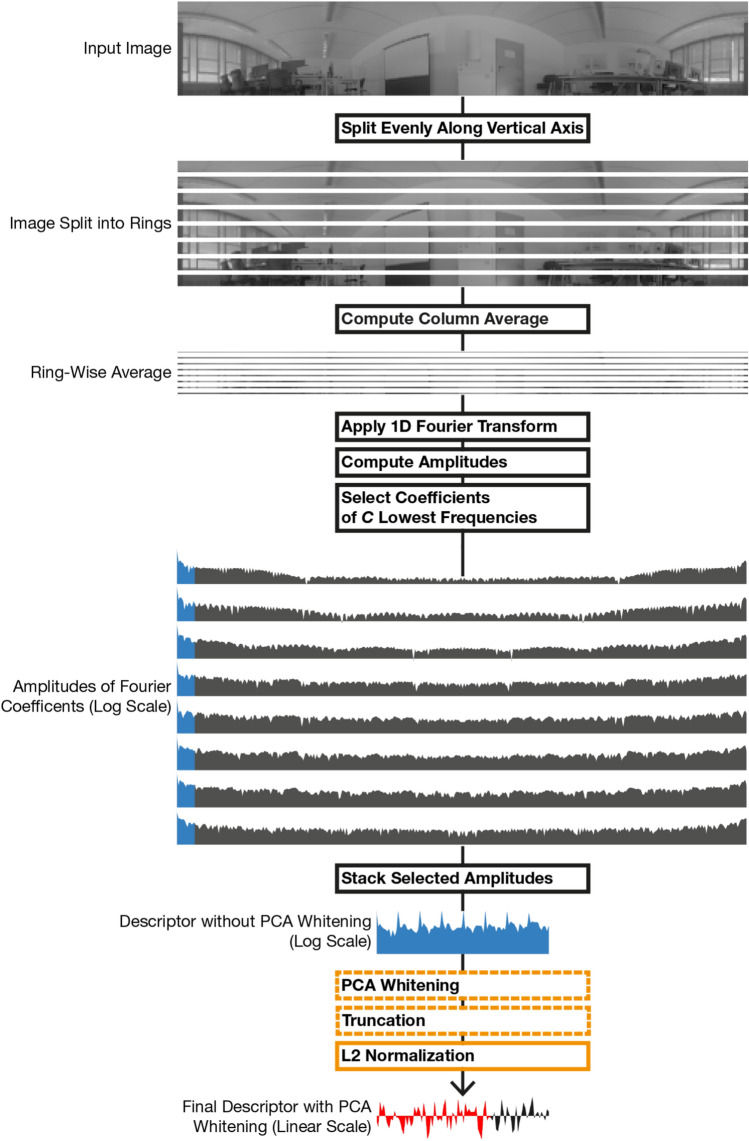
Overview of Fourier signature computation steps (boxes) with intermediate results. For visualization purposes, we only split the input image into 8 rings. Parts of the algorithm introduced in this work are marked orange. Post-processing steps with a dashed border are optional. When standardization is applied, it replaces PCA whitening and truncation. Amplitudes of the Fourier coefficients are shown in Log-Space. The 12 selected amplitudes of each ring are marked blue. As an example, the final descriptor is truncated after the first 64 elements, retaining two thirds of its original length (marked red).

We now introduce the Fourier signature algorithm as originally proposed by Menegatti et al.^2^ and with modifications by Gerstmayr-Hillen et al.^19^. An overview of computation steps, including post-processing with PCA whitening, and examples of intermediate results are shown in Fig. 3.

Computing a Fourier signature without PCA whitening follows these steps:

1. The panoramic image is split into a fixed number of rings with equal height.
2. We take the column-wise average of every ring, reducing them to a height of 1 px. This adaptation was introduced by Gerstmayr-Hillen et al.^19^; the original method^2^ uses a pixel-wise split.
3. The 1D Fourier transform is applied to each ring and the amplitude $|F(f)|$ is computed from the coefficients $F(f)$. Then, only the amplitudes of a fixed number of the lowest frequencies are retained.
4. The amplitude vectors of individual rings are then stacked to get the global image descriptor.
5. We introduce L2 normalization of the final descriptor, because we observe a general improvement of the recall over the unnormalized variant (data not shown).

Results for Fourier signatures without post-processing include L2 normalization.

### Descriptor post-processing

We extend the base Fourier signature algorithm with PCA whitening to rescale and decorrelate the descriptor entries. Additionally, we evaluate standardization, which also rescales the descriptor entries, but retains correlations. As a prerequisite for both methods, we calculate the unnormalized Fourier signatures for all $N$ images in the training dataset $\boldsymbol{x}_i \in \{\boldsymbol{x}_1,\dots ,\boldsymbol{x}_N\}$ ahead of time. At runtime of the VPR system, we build upon the unnormalized descriptor corresponding to the $i$-th visited place, $\boldsymbol{v}_i$, and the unnormalized descriptor of the current view $\boldsymbol{c}$.

#### PCA whitening

We follow the description of PCA whitening by Bishop^20^, starting with the calculation of a biased estimate of the covariance matrix $\boldsymbol{S} = \frac{1}{N}\sum _{i=1}^{N}(\boldsymbol{x}_i-\boldsymbol{\bar{x}})(\boldsymbol{x}_i-\boldsymbol{\bar{x}})^\top$. Here, $\boldsymbol{\bar{x}}$ refers to the mean $\boldsymbol{\bar{x}} = \frac{1}{N}\sum _{i=1}^N \boldsymbol{x}_i$. The $D$ principal variances and principal components then correspond to the eigenvalues $\lambda _i$ and eigenvectors $\boldsymbol{u}_i$ of $\boldsymbol{S}$, respectively, with $i \in \{1,\dots ,D\}$. Next, we create $\boldsymbol{L}$ as a diagonal matrix of the eigenvalues $\lambda _i$, and $\boldsymbol{U} = [\boldsymbol{u}_1|...|\boldsymbol{u_D}]$ as a square matrix containing all $D$ eigenvectors as columns. The eigenvalues in $\boldsymbol{L}$ and their corresponding eigenvectors in $\boldsymbol{U}$ are sorted such that $\lambda _1 \ge \lambda _i \ge \dots \ge \lambda _D$.

In this work, we compute $\boldsymbol{L}$ and $\boldsymbol{U}$ ahead of time and use them to whiten unseen data using a separate test dataset. Augmenting the training data with images of visited places during runtime may improve the VPR quality further. To this end, PCA statistics may be updated incrementally as described by Ross et al.^25^ to avoid managing a growing training dataset and re-computation of the full PCA.

When exploring the environment at runtime of the VPR system, we successively build a set of processed (i.e., whitened and normalized) descriptors of visited places $\boldsymbol{v}_{i,wn} \in \{\boldsymbol{v}_{1,wn},\dots ,\boldsymbol{v}_{M,wn}\}$. When a place is first visited, we compute $\boldsymbol{v}_{i}$, whiten it with $\boldsymbol{v}_{i,w} = \boldsymbol{L}^{-\frac{1}{2}}\boldsymbol{U}^\top (\boldsymbol{v}_i-\boldsymbol{\bar{x}})$, and apply L2 normalization to yield $\boldsymbol{v}_{i,wn} = \frac{\boldsymbol{v}_{i,w}}{||\boldsymbol{v}_{i,w}||_2}$. At retrieval time, the unnormalized descriptor of the current view $\boldsymbol{c}$ is processed the same way, which results in $\boldsymbol{c}_{wn}$.

The entries in descriptors $\boldsymbol{v}_{i,wn}$ and $\boldsymbol{c}_{wn}$ follow the ordering imposed on the eigenvalues in $\boldsymbol{L}$ based on the magnitude of the corresponding eigenvalues. This allows compression of the whitened descriptors by truncation after the first $T$ entries. In “Descriptor Size”, we show that $T=448$ is a suitable choice for the investigated datasets.

#### Standardization

To standardize Fourier signatures, we compute the mean $\boldsymbol{\bar{x}}$ and standard deviation $\boldsymbol{\sigma } = \sqrt{\frac{1}{N}\sum _{i=1}^{D} (\boldsymbol{x}_i-\bar{\boldsymbol{x}})^2}$ of unnormalized descriptors in the training dataset. At runtime, the processing of Fourier signatures follows the procedure as described for PCA whitening: We build a set of processed descriptors of visited places $\boldsymbol{v}_{i,sn} \in \{\boldsymbol{v}_{1,sn},\dots ,\boldsymbol{v}_{M,sn}\}$ and compute $\boldsymbol{v}_{i,s} = \frac{\boldsymbol{v}_i-\boldsymbol{\bar{x}}}{\boldsymbol{\sigma }}$ before applying L2 normalization $\boldsymbol{v}_{i,sn} = \frac{\boldsymbol{v}_{i,s}}{||\boldsymbol{v}_{i,s}||_2}$. The standardized and normalized descriptor of the current view $\boldsymbol{c}_{sn}$ is created the same way.

In contrast to PCA whitening, standardization does not rotate descriptors using the matrix of eigenvectors $\boldsymbol{U}$. Therefore, the standardized descriptor entries are not ordered by the principal variances $\lambda _i$ and compression by truncation is not applicable.

### Parameters

The retrieval quality of Fourier signatures can vary substantially depending on the choice of parameters. In the following, we establish our choice of the number of retained coefficients, the similarity functions, the number of image splits, and the truncation of the final descriptor. To this end, we evaluate possible choices using the average recall@10 on the training dataset.

#### Retained coefficients

We follow Horst and Möller^18^ and construct the Fourier signature from the coefficients of the 12 lowest frequencies. This choice is retained for all following experiments.

#### Similarity functions

**Fig. 4:**
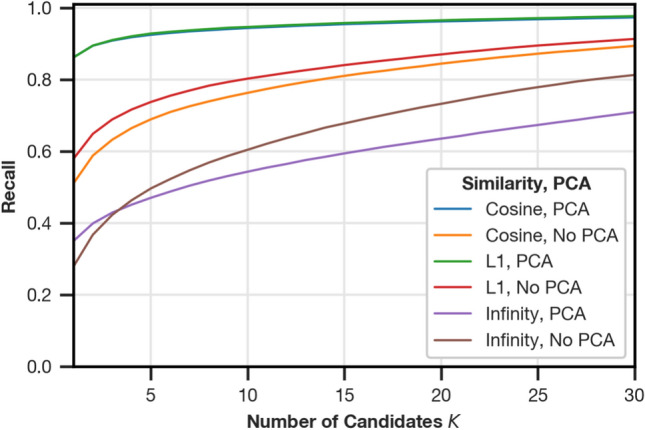
Recall for choices of the similarity function with and without PCA whitening as a post-processing step.

During image retrieval, descriptors are compared using a similarity function. We evaluate possible choices based on the L1 norm $S_{L1}(\boldsymbol{a}, \boldsymbol{b}) = -||\boldsymbol{a}-\boldsymbol{b}||_1$ suggested by Menegatti et al.^2^, the infinity norm $S_{\infty }(\boldsymbol{a}, \boldsymbol{b}) = -||\boldsymbol{a}-\boldsymbol{b}||_\infty$, $||\boldsymbol{x}||_\infty = \max (|x_1|, ..., |x_n|)$ adapted from Horst and Möller^18^, and the cosine similarity $S_{C}(\boldsymbol{a}, \boldsymbol{b}) = \boldsymbol{a}\cdot \boldsymbol{b}$ for Fourier signatures with and without PCA whitening. Note that using a similarity function based on the L2 norm ($S_{L2}(\boldsymbol{a}, \boldsymbol{b}) = -||\boldsymbol{a}-\boldsymbol{b}||_2$) is also possible, but yields the same ranking as the cosine similarity, because compared vectors are normalized. For the comparison of similarity functions, we set the number of image splits to 64; see “Number of image splits” for an independent evaluation of this parameter. The results are shown in Fig. 4.

For Fourier signatures without PCA whitening, $S_{L1}$ offers the best recall, while $S_C$ and $S_{L1}$ are good choices when PCA whitening is applied. To facilitate comparison between VPR with and without post-processing, we choose $S_{L1}$ for both methods.

#### Number of image splits

**Fig. 5:**
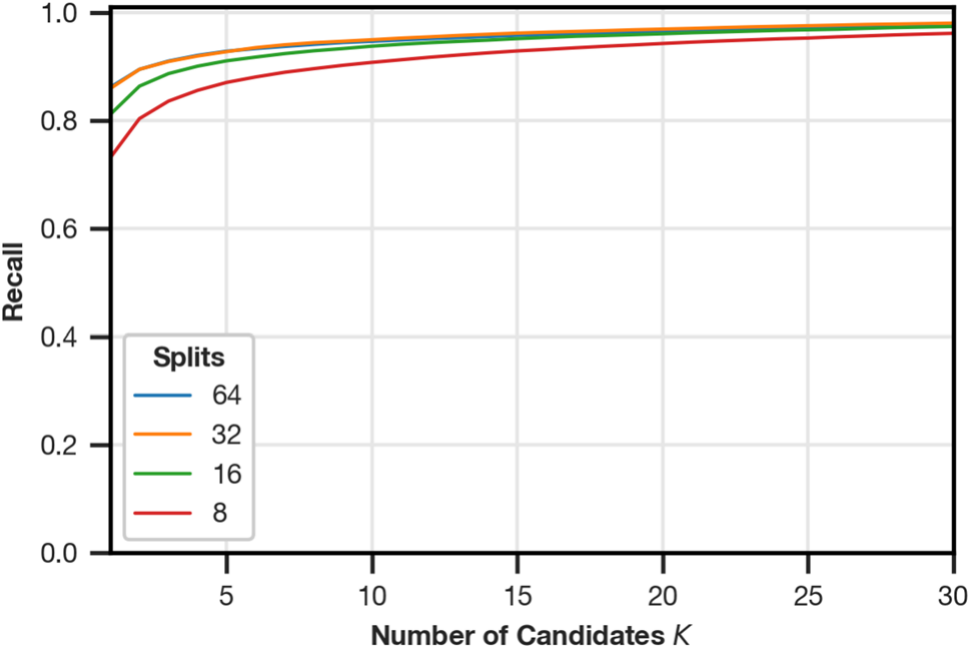
Recall for Fourier signatures with PCA whitening and the L1-based similarity function (see “Similarity functions”) with varying numbers of image splits.

To select the number of image splits, we evaluate the recall@10 with PCA whitening and the $S_{L1}$ similarity function for the splits 64, 32, 16, and 8 (see Fig. 5).

Both 32 and 64 splits offer the best recall@10. For the following experiments, we choose 64 splits, which is methodologically closest to the original algorithm proposed by Menegatti et al.^2^.

#### Descriptor size

Compressing the final descriptor by truncating it to a fixed size is only possible if PCA whitening is applied. To allow comparability of methods, we retain the maximum descriptor size of 768 and investigate truncation separately (see “Compression”4.4).

### AnyLoc

Keetha et al.^4^ evaluate vision foundation models with multiple aggregation methods for local features for a range of VPR settings. They propose to use features of the foundation model without fine-tuning; instead, domain adaptation is deferred to the aggregation method. Following their work, the model with the best recall across datasets is based on the DINOv2^26^ vision transformer and VLAD aggregation, which we select for further testing on our datasets and refer to as *AnyLoc*.

We use the implementation of AnyLoc for Torch Hub provided by the authors (https://github.com/AnyLoc/DINO) and create new VLAD centroids using our training dataset. This version uses the largest available DINOv2 model, ViT-g/14 (without registers). As local features, the *values* of the attention layer of block 32 are used. The dimensionality of the embeddings is 1536. VLAD then uses 32 centroids for clustering, resulting in raw descriptors of size 49152. Following the suggestion of Keetha et al., we then apply PCA and whitening. To this end, we use the PCA implementation of scikit-learn^27^ with a target descriptor dimension of 4096. The PCA statistics are computed using the training partition.

Note that the size of input images for AnyLoc during training and validation must be evenly divisible by 14, corresponding to the token size of DINOv2. To conform, we upscale dataset images from 64 $\times$ 384 px to 70 $\times$ 420 px (height $\times$ width).

## Results

In the following, we first show that PCA whitening offers the best recall on the test partition out of the tested post-processing methods. Then, we analyze the VPR quality for individual settings and the relation of the physical distance between capture points and the descriptor similarity. Next, we test the VPR quality for increasing levels of descriptor compression. Finally, we compare recall and execution times of Fourier signatures and AnyLoc.

### Overall recall

**Fig. 6:**
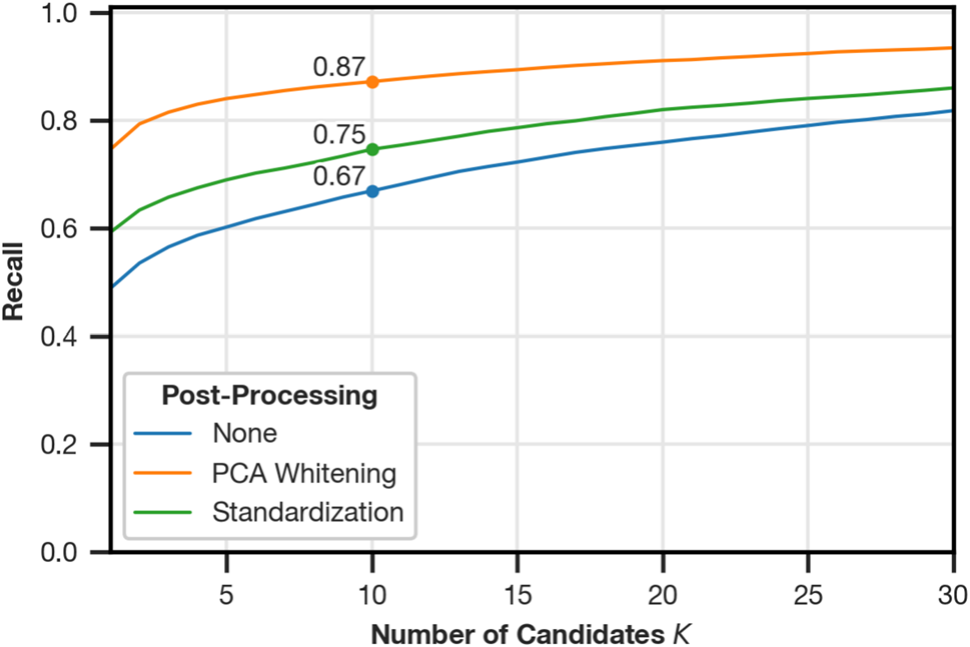
Comparison of the Recall@K between Fourier signatures without post-processing (“None”), standardization, and Fourier signatures with PCA whitening for the test partition of the dataset. The recall values are aggregated over all test settings as described in “Evaluation with Recall@K”. The marked spot for each post-processing method shows the recall for K=10.

Figure 6 shows the recall on the test partition of the dataset. While standardizing provides a benefit over the unprocessed Fourier signatures, PCA whitening further improves the recall for any tested $K$ by a similar margin. The amount of improvement shows that the statistics computed on the training partition are also suitable for unseen data.

### Setting-wise recall

**Fig. 7:**
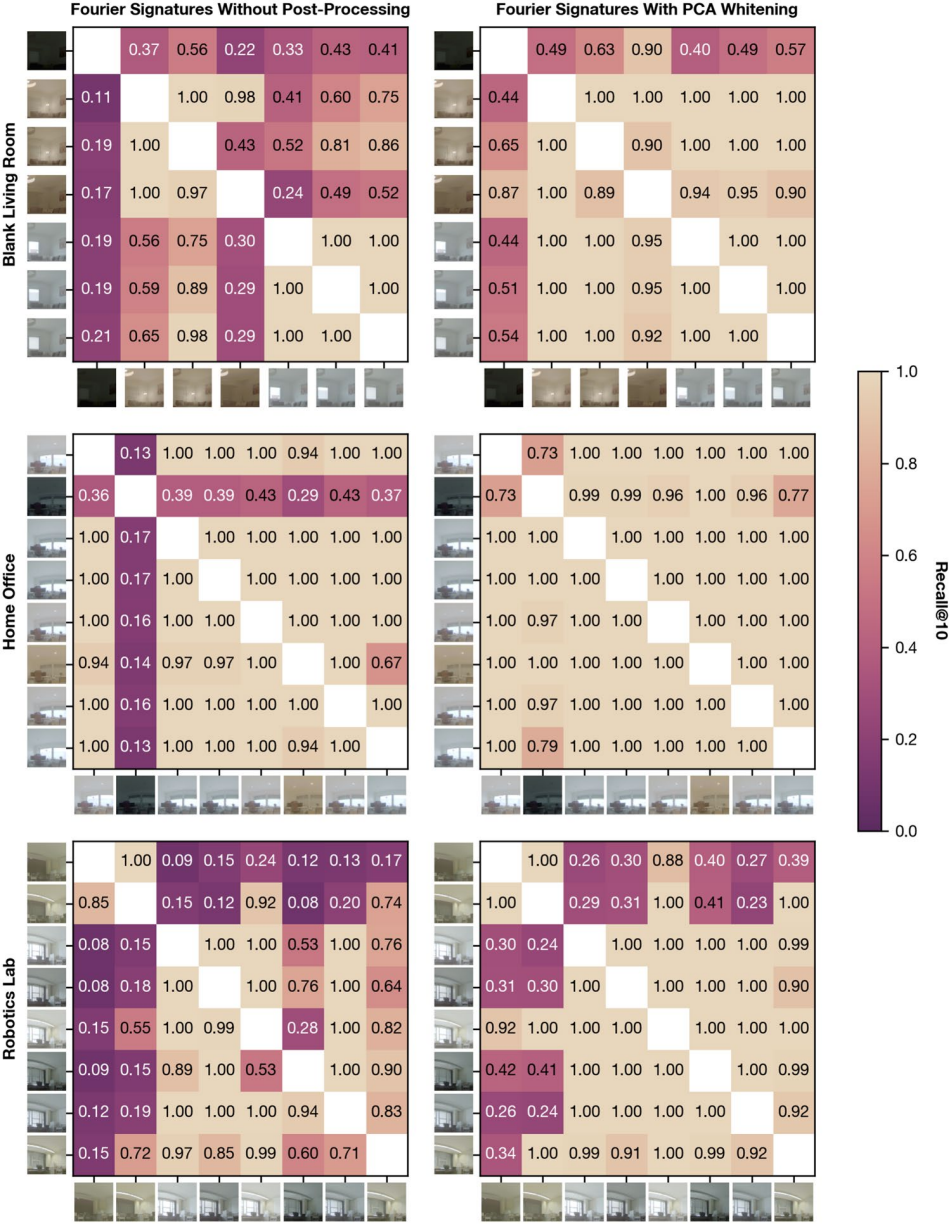
Recall for pairs of variants when retrieving K = 10 candidates for the settings Blank Living Room, Home Office, and Robotics Lab from the test partition of the dataset. The left plots show the recall for Fourier signatures without post-processing and results for Fourier signatures with PCA whitening are shown on the right. The ticks for each heatmap correspond to the variants. For each variant, a section of the panoramic image is shown akin to Fig. 2 to visualize the illumination of the environment.

Investigating the recall@10 for individual settings and variant pairs of the test partition in Fig. 7 confirms these improvements. Following this, it is reasonable to expect a meaningful improvement of VPR quality when applying PCA whitening over the unprocessed descriptors. Notable exceptions pose the dimly illuminated variant for the setting Blank Living Room and the variants with black curtains and artificial illumination for Robotics Lab. The former case shows the limits of the added illumination tolerance afforded by PCA whitening. However, for the latter case, the change in illumination in comparison to other settings seems moderate. Instead, we suspect that closing the curtains along one side of the room causes a portion of the image to change to a more homogeneous color, which reflects in the frequencies captured by the Fourier signatures, making place recognition based on the frequency spectrum challenging.

### Relation of physical distance to descriptor similarity

**Fig. 8:**
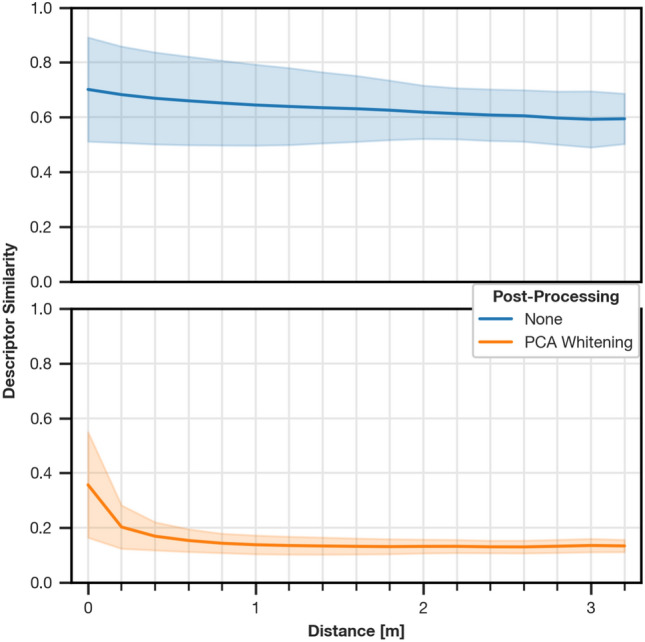
Distribution of $S_{L1}$ similarities between descriptors in relation to the physical, planar distance between capture points. To estimate the distribution, we compute similarities between all images in the test partition (excluding variant pairs with same illumination) along with their physical distance from ground truth data. Then, similarities are grouped into bins of 20 cm width. Bin edges are placed at half distance between ticks along the x axis (e.g., the 20 cm bin contains all distances in the half-open interval including 10 cm and excluding 30 cm). For all bins, we show the mean as a solid line and the standard deviation as a shaded area. Similarity values are normalized to the range $[0,1]$.

Relating the $S_{L1}$ similarity between descriptor pairs to the physical distance of their capture points in Fig. 8 shows a smaller variance of the similarity for whitened descriptors, which indicates an increase in overall robustness. We also observe a larger difference between similarities at the same position and neighboring grid points, which helps to distinguish images taken at the same position from images taken at close positions.

### Compression

**Fig. 9:**
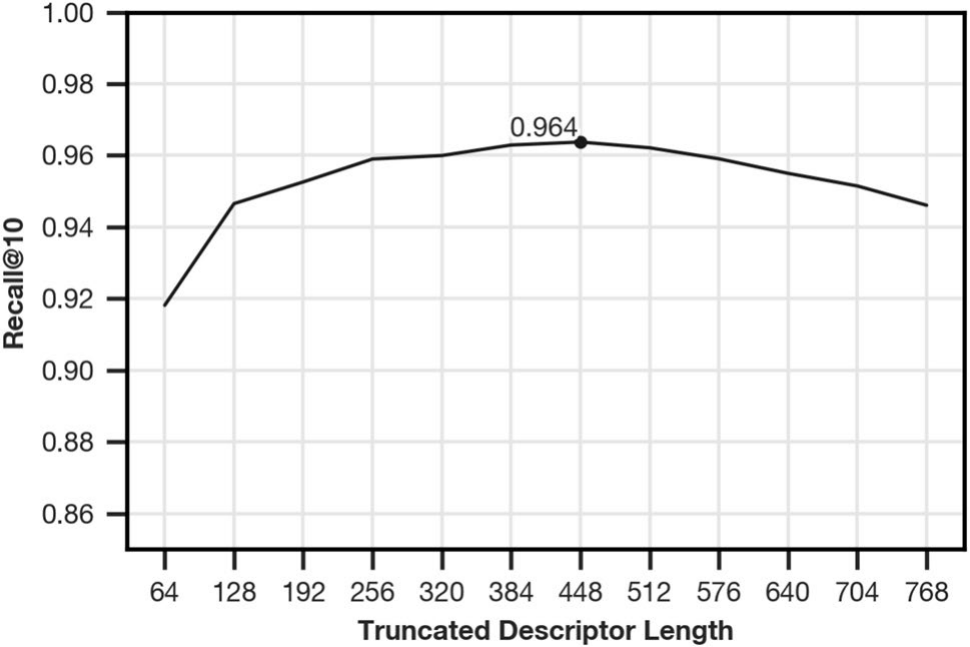
Recall@10 for truncated Fourier signatures in steps of 64 from 64 to the full descriptor size, 768. This parameter must be selected once before using the algorithm in the field; consequently, we evaluate on the training partition. All values refer to an average over settings and variant pairs as described in “Evaluation with Recall@K”.

Using PCA whitening allows compression by truncation of the descriptor, with possible degradation of the VPR quality. Splitting the image into 64 rows and retaining 12 coefficients results in a raw descriptor size of 768. We evaluate truncation in steps of 64 up to a remaining size of 64. As shown in Fig. 9, the recall@10 on the training partition only drops off meaningfully for a remaining size of 128 and shorter. The optimal descriptor size for the training dataset is 448, which offers the best recall@10 of about 0.964. All values are rounded to three decimal places.

### Recall and execution times compared to AnyLoc

**Fig. 10:**
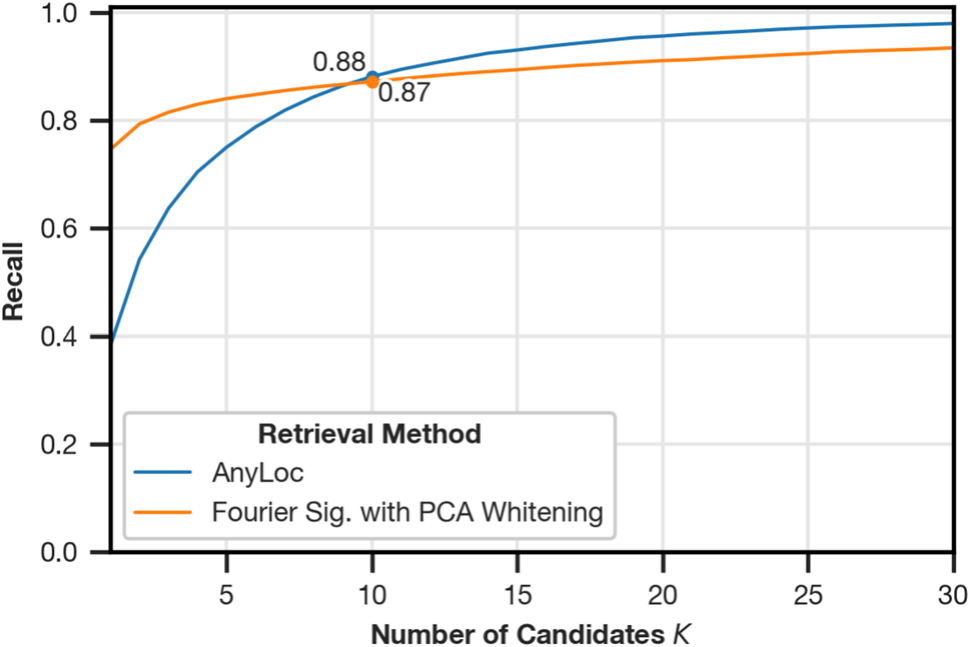
Comparison of the recall for variations of $K$ between AnyLoc^4^ and Fourier signatures with PCA Whitening. The recall values are aggregated over all test settings as described in “Evaluation with Recall@K”. The marked spot for each method shows the recall for K=10.

Figure 10 shows the comparison between the recall of AnyLoc and Fourier signatures with PCA whitening on the test partition. For $K<9$, Fourier signatures offer better recall while AnyLoc offers better VPR quality for $K>9$. Near the point of intersection at $K=9$, the recall of both methods is similar.

Additionally, we compare execution times of Fourier signatures and AnyLoc in Table 1. Measurements are taken on a desktop computer with an Intel Core i9-10980XE CPU and an NVIDIA RTX 3090 GPU with 24 GB of VRAM. The system runs Linux Kernel 6.9.7, Nvidia driver version 555.58.02, CUDA 12.8, and Python 3.12.4. To run AnyLoc, we use PyTorch 2.9.0 with torchvision 0.24.0. To speed up inference, we compile the AnyLoc model using TensorRT 10.13.0.35 and the torch-tensorrt 2.9.0 package. The inference model is configured to use a batch size of 1 and single precision floating points.

Fourier signatures with PCA whitening is implemented using the fast Fourier transform and matrix operations of NumPy 1.26.4. Here, no GPU acceleration is used.

The execution time is measured using the *default_timer* of the Python *timeit* module and refers to computing the descriptor for a single, preprocessed image. Statistics of the results shown in Table 1 are computed for 10000 samples. For AnyLoc, the times exclude upscaling (see “AnyLoc”), but include copying the image to GPU memory, copying the raw descriptor back to the host memory, and applying the PCA whitening proposed by the authors^4^.

**Table 1:** Comparison of execution times of the presented methods. All values are in milliseconds and rounded to 5 decimal places.

Method	Average time (ms)	Standard dev. (ms)	Max. time (ms)
Fourier signatures	0.00336	0.00074	0.00106
AnyLoc	0.04897	0.00464	0.10635

## Discussion

The improvement of the recall of Fourier signatures with post-processing can be attributed to two separate effects, represented by the rescaling of descriptor entries through standardization and the additional decorrelation by PCA whitening. Additionally, the comparison with AnyLoc shows that Fourier signatures can be competitive with modern deep-learning-based methods.

### Rescaling

**Fig. 11:**
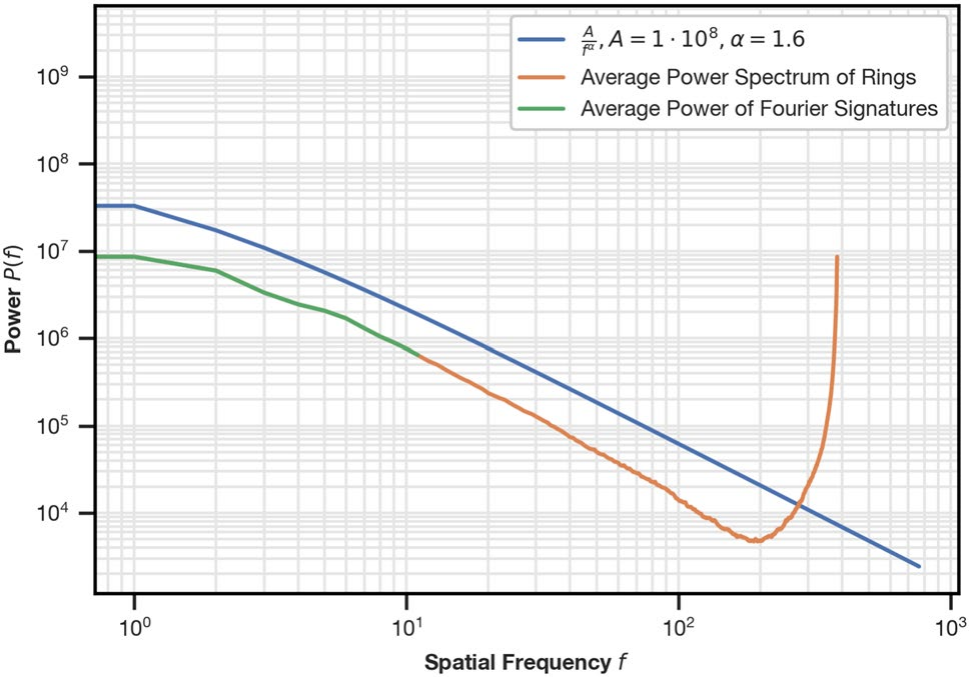
Average power spectrum of the rings of panoramic images of the test partition (orange) and the section used in Fourier signatures (green). Because Fourier signatures use a subset of the full power spectrum, the green curve overlaps the orange curve exactly. The power law model $P(f) \propto \frac{1}{f^\alpha }$ with example parameters is shown for reference.

Multiple authors^28–33^ observed that the power spectrum $P(f) = |F(f)|^2$ of the Fourier coefficients $F(f)$ of natural images follows a power law $P(f) \propto \frac{1}{f^\alpha }$. The exponent $\alpha$ varies on a per-image basis and is expected^33^ to be around 2. This model is valid for any orientation of the observed frequencies^33^ and therefore also applies to 1D image rings of natural panoramic images (see Fig. 11).

The entries of Fourier signatures follow the same model, causing large differences in scaling between the amplitudes of individual frequencies. Without rescaling, the similarity between image descriptors is therefore determined by only a small subset of the observed frequencies. We attribute the VPR quality improvement afforded by standardization to its rescaling of the descriptor entries to unit variance.

### Correlation after standardization

**Fig. 12:**
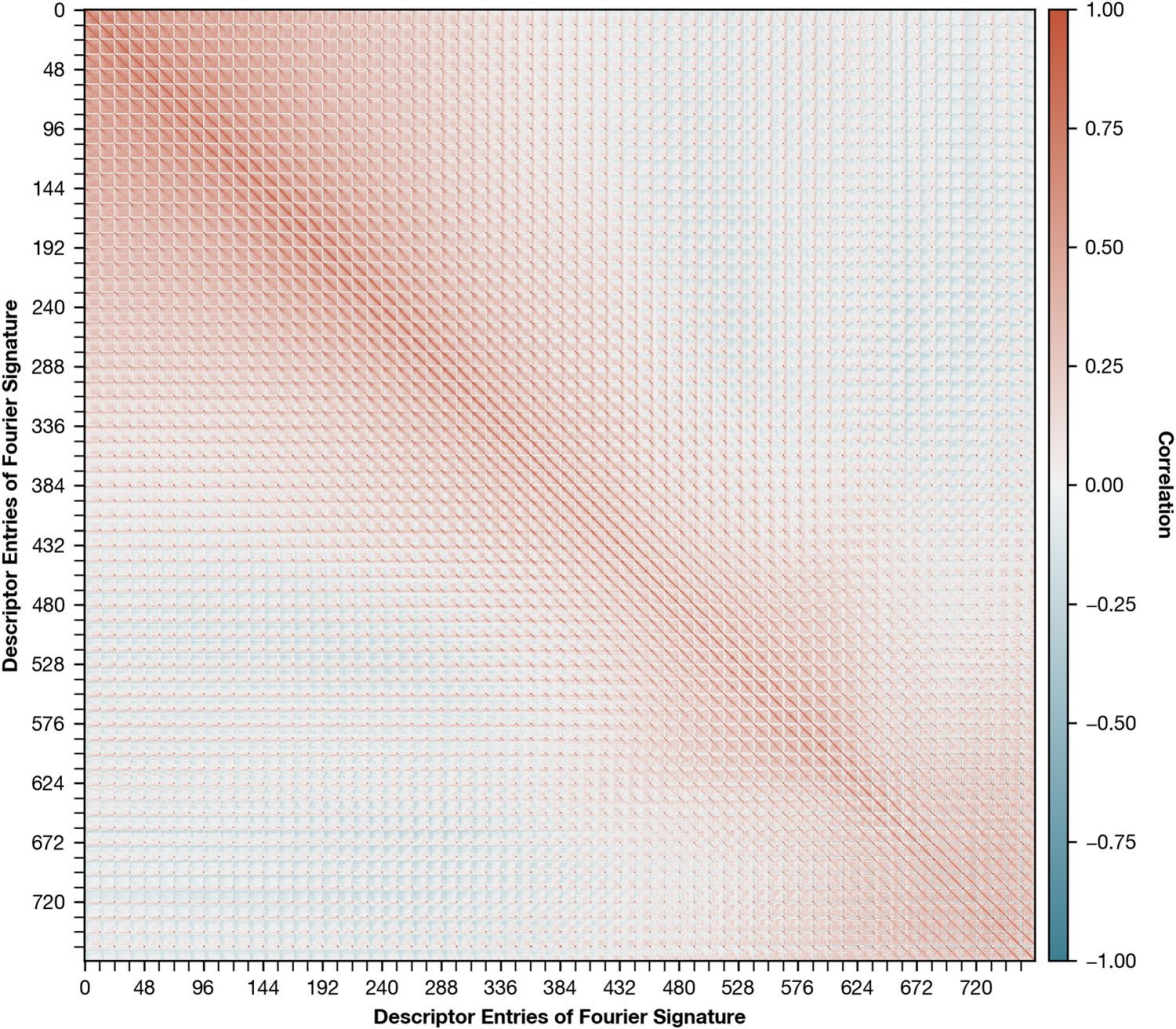
Covariance matrix of Fourier signatures of the training partition after standardization, showing correlation between descriptor entries. By design, the covariance of whitened descriptors is equal to the identity matrix^20^.

Figure 12 shows the correlation of descriptor entries after standardization. The high correlation results in prominent off-diagonals and is caused by the similarity of neighboring image rows and their amplitude spectra. We also observe a block-like structure of positive correlations, especially for descriptor entries with low indices. This is likely caused by rows with low indices showing ceilings with homogeneous colors, which results in a spectrum of similar frequencies. Finally, the amplitude of the zero frequency (corresponding to the mean of the image row) is highly correlated across frequency spectra of different rows but does not correlate with the amplitudes of other frequencies. This results in a dot-pattern, with the spacing corresponding to the number of coefficients selected for the Fourier signature (in this work: 12, see “Retained coefficients”). Conclusively, the correlations between descriptor entries can be explained either by the general image structure or the design of the descriptor, meaning that they do not contribute to describing the place the image was taken at and should be removed.

Compared to standardization, PCA whitening also provides the desired rescaling, but also decorrelates the vector entries. In removing the correlations, PCA whitening creates more discriminative Fourier signatures and allows for effective descriptor compression (see “Compression”).

### Comparison with AnyLoc

In “Recall and execution times compared to AnyLoc”, we apply AnyLoc to our test dataset and observe that the domain adaptation works well despite the change to the panoramic image format. However, Fourier signatures with PCA whitening offers better recall for $K < 9$ and is still competitive at around $K=10$. At the same time, it requires substantially fewer resources than AnyLoc as shown in Table 1. This is expected: both algorithms employ PCA whitening, but AnyLoc is based on a ViT-g/14 neural network with 1.1 billion parameters^4,26^, while the configured Fourier signatures computes 64 1D fast Fourier transforms on 384 pixel wide sections. Our results indicate that Fourier signatures with PCA whitening poses a light-weight alternative for panoramic images.

## Conclusion

We conclude that the quality of VPR of Fourier signatures in situations with varying illumination can be improved by applying PCA whitening as post-processing to the global image descriptors. Here, we demonstrated the effectiveness for a range of common illumination changes. Limits of the method include extreme illumination conditions and specific changes of large parts of the observed environment. Using standardization as an alternative post-processing method, we attribute the improvement of the recall to two effects of PCA whitening. First, the rescaling of the descriptor entries removes differences in scaling, and second, its decorrelation removes redundant information introduced by the image structure and descriptor design.

We also showed that Fourier signatures with PCA whitening are competitive with AnyLoc, a modern deep-learning-based image retrieval method, while requiring substantially fewer resources.

## Data Availability

The preprocessed image database and source code are available at https://gitlab.ub.uni-bielefeld.de/loffermann/fourierpca.
